# Elite Youth Soccer Players’ Sources and Types of Soccer Confidence

**DOI:** 10.3390/sports9110146

**Published:** 2021-10-22

**Authors:** Iain Greenlees, Aimee Parr, Sarah Murray, Esther Burkitt

**Affiliations:** 1 Institute of Sport, Nursing and Allied Health, University of Chichester, Chichester PO19 6PE, UK; aparr1@chialumni.ac.uk (A.P.); s.murray@chi.ac.uk (S.M.); 2 Department of Psychology and Counselling, University of Chichester, Chichester PO19 6PE, UK; e.burkitt@chi.ac.uk

**Keywords:** self-confidence sources, self-efficacy, elite youth athletes, soccer

## Abstract

Sport confidence is a psychological characteristic considered vital for youth soccer players to possess. However, only limited research has explored the types and sources of sport confidence important to elite youth performers in professional soccer academies. Semi-structured interviews were conducted with 11 academy footballers (aged 10 or 11). Abductive hierarchical content analysis identified types of confidence to include achievement, skill execution, psychological factors, superiority to opposition and tactical awareness. Key sources of confidence identified by players were performance accomplishments, coaching, social support, and preparation. Even though the dimensions reported were similar to previous research, a number of unique sub-themes of confidence sources emerged, including pre-training/competition emotions, coach and team-mate feedback. The results demonstrate the importance of considering maturation levels and context when seeking to understand and develop confidence in youth performers.

## 1. Introduction

Numerous factors (physiological, technical, tactical, sociological and psychological) have been proposed to be important predictors of talent development and long-term playing success in soccer [1]. One psychological factor that appears pivotal is self-confidence [1]. Within high level youth soccer, confidence has been implicated as a central requirement not only for development and the transition through youth to elite performance [2,3] but also for the maintenance of positive psychological well-being [4]. Despite this, we still know relatively little about the determinants of confidence in high-level youth sport, yet alone in soccer.

In sport, understanding of confidence has been largely informed by the work of Bandura’s [5] concept of self-efficacy and by the work of Vealey and colleagues [6,7,8]. These approaches conceptualise confidence as a dynamic psychological state influenced by a range of personal, demographic and environmental factors. In their general model of sport confidence, Vealey and Chase [8] propose that sport-confidence (one’s belief that one can be successful in a chosen sport) is a multidimensional construct made up of confidence in executing skills (SC—physical skills and training), decision-making and concentration (SC—cognitive efficiency) and dealing with setbacks (SC—resiliency). Vealey and Chase [8] further propose nine sources of confidence nested within 3 domains (Achievement (including mastery or improvement of personal skills and demonstration of ability by showing others or demonstrating superior ability to opponents), Self-regulation (including physical and mental preparation and physical self-presentation) and Climate (including social support, vicarious experiences, coach’s leadership, environmental comfort and situational favourableness). Importantly, Vealey and Chase [8] propose that, rather than being universally important, the importance of each of the sources of confidence will differ across individuals and contexts.

The principles of the model have received broad support in the literature. Hays, Maynard, Thomas and Bawden [9], in a study of world-class, adult performers, provided support for the multi-dimensionality of confidence types and sources but differed from Vealey and Chase’s [8] model in two ways. First, performers reported six, rather than three, types of confidence (skill execution, achievement, physical factors, psychological factors, superiority to opposition and tactical awareness). Second, whilst preparation, performance accomplishments, coaching, and social support all emerged as sources of confidence from the data, little evidence was found to support the role of vicarious experiences, physical self-presentation, environmental comfort or situational favourableness. In addition, Hays et al. [9] found that trust, competitive advantage and self-awareness emerged as sources of confidence information.

Even though this model and research provide some insight into sources of confidence in sport, extrapolating these findings to elite youth sport is problematic for a number of reasons. First, Vealey and Chase [8] stress that age influences the sources of confidence that are most relevant to an individual. Vealey et al. [7] argued that schemas concerning sources of confidence are developed during childhood and adolescence and so this age period may witness fundamental changes in sources of confidence. Research in youth sport settings (for reviews see [4,10]) has shown that, compared to adult and older adolescents, children utilise fewer sources of competence information, show less ability to integrate and differentiate between different sources of information, and rely more on concrete and external information sources (in the form of actual performance outcomes and coach/parent/peer feedback). Second, Vealey and Chase [8] also propose that organisational and cultural characteristics (such as expectation levels, the quantity and quality of feedback provided and the nature of the social relations) will also influence the types and sources of confidence that are most relevant to performers. Thus, whilst findings from adult performers [9] and from children engaged in youth sport [10] may not truly reflect the types and sources of confidence important to performers in the unique and rarefied environment of elite youth soccer.

In acknowledgement of the potential lack of generalisability from elite, adult performers to youth athletes, Thomas, Thrower, Lane and Thomas [11] explored types, sources and debilitators of confidence in elite (signed to the academy system of professional football clubs), adolescent soccer players. The ages of these players ranged from 12–15, with a mean of 14.28 years. Even though their results were broadly consistent with Vealey and Chase’s [8] and previous research [9] in terms of the types and sources of confidence reported, they also showed that there were differences in the number of sources of confidence reported and the importance of different sources of confidence. Specifically, whilst Thomas et al. [11] did find that performance accomplishments, preparation and social support were important sources of confidence, their results did not match the work of Hays et al. [9] or Vealey and Chase’s [8] model in demonstrating the importance of sources such as environmental comfort, situational favourableness, self-awareness or experience. This research demonstrates the need for more research to explore the distinct profiles of confidence types and sources across a range of populations and settings.

In light of this, the aim of the current research was to explore the types and sources of confidence of elite youth soccer players at the younger age range (10–11 year olds) than those used in Thomas et al.’s [11] research (early adolescents aged between 12 and 15). This age was chosen for three reasons. First, our sample represents an earlier stage of development than players included in Thomas et al.’s research. Whilst Thomas et al. sampled from the early adolescent stage, our players were in middle to late childhood according to established criteria [4]. This, theoretically, will entail different sources of confidence being used due to different self-perceptions and complexity of self-perceptions at this age [4]. Specifically, it could be predicted that our sample would rely on fewer sources of confidence, would rely on tangible achievements to gain confidence (e.g., scoring goals, being made captain) and would have a greater tendency to rely on others to interpret and evaluate their competence [12]. Second, research has highlighted the importance of confidence at this age for the development of youth soccer players [3]. Thus, it seems a particularly important age range to explore sources of confidence within. Third, the origins of this project were rooted in informal discussions with coaches at the soccer club involved which identified confidence in this age group as being particularly important to understand.

## 2. Materials and Methods

### 2.1. Philosophical Underpinning

A critical realist [13] philosophical perspective informed the current project. This perspective proposes that there is a reality that exists independently of subjective perceptions (ontological realism) but that access to this depends on fallible perceptions and subjective interpretations that are limited, incomplete and theory-dependent (epistemological relativism).

### 2.2. Context

Within English soccer, academies are run by professional clubs with the aim of producing professional players from the age of nine. Academies are classified from Category One to Four, with one being the highest level. Categorisation is determined by factors such as training facilities, productivity of the academy, welfare and development provisions. Places are highly prized and competitive but difficult to retain with regular rounds of re-selection [14] based on individual progression. The academy that this research was situated in considered itself a progressive academy with player development and welfare firmly at its heart. However, competition for places was high and, as a result, players (and their parents) were acutely aware of the need to impress coaches to retain their place in the academy.

### 2.3. Participants

In this case, 11 male academy players (aged between 10 and 12) from a Category One academy of an English Premiership club participated in the study. Participants were either in the under 11’s (9 players) or 12’s squad (two players) and all player positions (goalkeeper, defender, midfielder and forward) were represented. The average age of the participants was 11.57 years. Time spent at the academy ranged from one to four years. All participants self-reported as White-British.

### 2.4. Procedure

Ethical approval was provided by the Research Ethics Committee of the authors’ university. Following approval for the study being provided by the football club and relevant coaches, informed written consent was provided by parents. Then, all players in the targeted squads were provided with written information and a verbal summary of the project from the academy’s lead sport psychologist and asked if they would want to volunteer. We used a stratified purposeful sampling strategy [15] to select participants from those who volunteered. Participants were selected to represent a range of confidence levels, playing positions and years of experience. Written consent was provided by all participants prior to the data collection commencing. All interviews were conducted on a one-to-one basis in a quiet area of the football club’s training facilities and lasted between 31 and 51 min (M = 42 min).

#### Interview Guide

A semi-structured interview guide, based on information provided by Hays et al. [9], was developed. This guide was divided into five sections. First, introductory comments reminded participants of the study’s purpose and reinforced confidentiality and anonymity. Second, participants were asked general questions about self-confidence (e.g., “what does the term self-confidence mean to you?”). Similar to Hays et al., we did not provide a standardised definition of self-confidence as the aim was to develop greater knowledge of the participants’ perspectives. Next, participants were asked questions related to their perceptions of the types and sources of confidence within youth football. The fourth section of the interview asked about times of high and low confidence and situations in which they had experienced changes in confidence. The interview concluded with an invitation for the participants to provide further information concerning their confidence. Throughout the interview, clarification and elaboration probes were used to ensure that participants provided a rich and detailed response to the questions asked [16].

### 2.5. Data Analysis

In line with similar approaches used in research examining youth sport experiences (e.g., [2,11]), qualitative data were analysed using an abductive hierarchical content analysis. Here, the transcripts were examined and any meaningful units of information (such as key words or phrases) relating to the types and sources of confidence were identified. The first and second author independently listened to the recordings and read and re-read the transcripts to ensure familiarity with each script before any analysis took place. The second author then completed initial coding which was developed in discussion with the first author, who acted as a critical friend. After the inductive stage, we used a more deductive phase whereby the existing literature (e.g., [7,9,11]) was compared with our findings. The deductive phase was also used to prompt the use of terminology to increase consistency with previous research findings. Themes which did not fit any of the underlying dimensions were grouped together, sorted into common themes and subsequently labelled according to the meaning of the data. This approach permits the emergence of new raw-data themes (units of information) whilst ensuring that the theoretical frameworks underpinning the research retains a central position in the enquiry [2].

### 2.6. Enhancing Ontological Plausibility and Validity

In line with our critical realist perspective, and the writings of Maxwell [17], we accept the inherent fallibility of the research methods and analytical procedures that we have used but also took a number of steps to address threats to descriptive (the factual accuracy of the research account), interpretive (the extent to which our interpretations accurately reflect the participants’ experience) and theoretical (the extent to which our findings explain the experiences of participants) validity. In doing this, we were guided by the suggestions of Ronkainen and Wiltshire [18]. Specifically, to enhance descriptive validity the interviewer (second author) made notes following each interview and transcripts were checked by two authors. To enhance interpretive validity, we considered competing explanations of the evidence through the lead and second author independently coding the data before comparing interpretations, the use of critical friends, discussions with the participants’ coaches and sport psychologists to consider emerging themes and to offer reflections on our findings and, ultimately, via peer-review. The discussions with coaches and sport psychologists involved the presentation of research findings to coaches in a workshop that sought to discuss the findings and to consider ways in which the information could be used to inform practice. This confirmed our interpretations and also provided further support for the powerful role of moods in determining self-confidence. In addition, the second author kept a reflexive diary [19]. Diary entries were made after each interview allowing a focus on internal responses to being a researcher and emotional feelings in the process of data collection to be noted [20]. Finally, we argue that theoretical validity was enhanced as the research project was developed with the coaches in light of a perceived need to enhance their knowledge of self-confidence in academy soccer players and through the practical suggestions that were developed in light of our research findings.

## 3. Results

We present the results in two parts. First, we cover types of confidence and, second, we present sources of confidence. The data are presented in Table 1 and Table 2 and through the use of representative quotations (with pseudonyms used throughout). A fuller outline of the content analysis can also be found in Supplementary Materials Tables S1 and S2. Even though we recognise the limitations of response frequencies, we present them to facilitate comparison with previous findings [9,11].

### 3.1. Types of Confidence

In this case, 22 raw-data themes were categorized into seven global dimensions. These related to skill execution, achievement, psychological factors, superiority to opposition, tactical awareness and athlete specific factors. These are summarised in Table 1 below.

#### 3.1.1. Skill Execution

Nine of the 11 athletes found skill execution to be a type of confidence important to them, referring to their belief in their own ability to execute skills in soccer. This often included specific skills during a match. As Bailey put it “I think I’m quite good at passing. I’m not, I’m not the best at shooting, so I just, I don’t shoot, I just assist people”.

#### 3.1.2. Achievement

Six athletes identified achievement. This concerned belief that certain outcomes or performance-based skills could be achieved. Athletes talked about the need to be confident in their own ability: “You have to be confident in yourself and your ability to play football and like confident that you can actually achieve things.” (Will). Only Felix mentioned achievement in outcomes, highlighting his need to be confident in “scoring a good goal and winning”.

#### 3.1.3. Psychological Factors

Three athletes spoke of their need to be confident of psychological factors, including having a positive mind set for soccer. “You have to have a good mentality, like if you have a bad mentality then your confidence won’t be like, it will just drop, it will keep dropping.” (Freddie).

#### 3.1.4. Superiority to Opposition

Three athletes identified superiority to an opponent as a type of confidence, with one player needing to feel confident in “beating players with skills and dribbles.” (Will).

#### 3.1.5. Tactical Awareness

Tactical awareness was identified as a type of confidence by only two athletes. This referred to being confident about their own tactical ability, such as ball placement or decision making.

#### 3.1.6. Athlete Specific Factors

Two athletes identified unique types of confidence which were individual to them. This included having confidence in their communication with team-mates and having confidence in team-mates and their soccer ability.

### 3.2. Sources of Confidence

Inductive analysis of confidence sources yielded 117 raw-data themes. We conceptualised these into 24 lower order and sub-themes and 15 higher order themes. These are summarised in Table 2 below.

#### 3.2.1. Performance Accomplishments

This consisted of three higher order themes, competition accomplishments, training accomplishments and other accomplishments. All 11 players highlighted performing successfully and mastering specific skills, both in matches and training. Competition accomplishments included in-game performance elements, being selected, gaining awards and general wins and losses. For example, Dan reported increased confidence when he “scored a great goal from like the half-way line then that gave me confidence and I done this good run, beat 2 players and scored again and I set up another goal” (competition performance accomplishment) whilst Luke stated:

“I got an assist about 30 s as soon as I came on cos someone got injured and then I kept doing good passes and keeping the ball and then it made me feel happy so then I could like play better and like forget anything bad that happened in that game.”

A range of training accomplishment sub-themes were apparent. These included skill development and mastery, general performance levels, displaying superiority to others and being given the captaincy. The importance of training performance accomplishments was encapsulated by Bradley when he commented:

“I did well in training so like I kept trying it and then whenever I try I get better at it and then you get more confident doing it. If I keep practicing it and get really good at it then I can like do it in matches and then it will like work all the time and then I will just do it all the time.”

Players also identified how achieving success in school level football and in other sports could enhance their feelings of soccer-specific confidence.

#### 3.2.2. Coaching

All players identified the coach as a confidence source. Recognition, praise/criticism, attention/feedback and athlete-handling were identified as important. Praise and criticism appeared to be a big contributor, with Bradley stating, “when they encourage you to do stuff and like praise you it boosts it so much cos you know you’ve achieved something” and a second stating:

“If like you get more praise, if you keep getting praise in training and you are doing everything right they’ll give you more praise and it will make you play better and then your confidence will rise but if you’re playing bad and they’re like moaning at you it can affect some players.”

Recognition from coaches played a role in increasing confidence levels. Bailey spoke of recognition within the academy when he said “Getting a two-year contract I think, cos then you know that you’ve done it, like then you know that you’re good” whilst others spoke of recognition in terms of selection to their preferred position or being selected to be the captain. As Freddie put it:

“We were playing at the xxxxx [home stadium] and whoever got trainer of the night that Friday would be captain and I go through the training session and get trainer of the night and I’m, I walk out onto the xxxxxx with the captain’s armband on and I just felt really good. Wearing the captain’s arm band on the actual xxxxxxxx [home stadium] makes you feel really good!”

Whilst performance accomplishments were important, a number of the performers relied on their coaches to inform them when a performance was good and to recognise, beyond obvious markers of good play such as goals scored, when they had performed well. As Felix commented “Yeah, cos if I think that I’m not playing well at all then someone (coach) comes over to me and say what I have done well and then I feel more confident”.

#### 3.2.3. Social Support

Ten players discussed social support. This consisted of the higher order themes of family, team-mates, school/friends and external others (other coaches and spectators) support. Within the higher order family support theme, lower-order themes of atmosphere, feedback and support were seen. Having family support was seen as important, as was gaining positive feedback from family members with Max stating “if I’ve played well then my dad gives me more confidence and he says ‘oh you’ve done well.’”. The participants reported both positive, negative and neutral impacts of parental feedback on their confidence, with Ashley explaining “If I’ve played well then like my dad gives me more confidence and he says oh you’ve done well and if my mums says I played well and I have played well then it sort of stays the same”. For some, the negative impact of parental feedback and criticism appeared to be exceptionally strong. For example, Max said “Like sometimes my dad will moan at me [pause], a lot. He won’t stop until I’m like almost in tears and that will affect my confidence” whilst Jordan commented:

“They [referring to parents] say it in kind of a bad way and they should just say “that wasn’t very good today, try and do better tomorrow” but they say “that was terrible, if you’re gonna carry on doing that you’re gonna get kicked, there’s better boys out there who want to be there and it looks like when you’re on the pitch you don’t even wanna be there.”

Further social support was found from teammates and team support. The dangers of a negative, critical team atmosphere was highlighted frequently, with Bailey stating:

“If you didn’t have team work then you would just be like running with the ball, no one’s passing, everyone’s moaning at each other and everyone’s confidence will be down.”

Furthermore, Sam commented:

“Sometimes my teammates don’t give me as much confidence because sometimes they put me down. Especially coming to the end of the season it’s really hard because they, they’re fighting for the same position that you are so they are trying to knock you down.”

Finally, participants also mentioned the important role of people outside of the immediate family and academy in developing and maintaining their confidence levels. References were made to school friends, teachers and opposition and neutral spectators in developing and reducing confidence.

#### 3.2.4. Preparation

This consisted of two higher-order themes, physical/mental preparation and holistic preparation. Whilst physical/mental preparation encompassed a range of sources (e.g., having warmed up well), we were struck by the seeming importance attached to what we referred to as holistic preparation. Specifically, the players commonly reported that general life-events and hassles had an important influence on their confidence (both negatively and positively) that seemed to reflect the effect of general mood on confidence. The following quotes illustrate this:

“Home, so say somethings going on at home, it would make it [referring to confidence] lower. So say if something is going on in school maybe it’ll make it lower or something’s… like you’re doing well in school, you’re doing well in something it will make it higher (Will).”

“Well I want to do well most of the time but sometimes like I wake up and I’m a bit like I don’t really feel like playing football and I don’t really have a good game. Yeah, it makes me… cos I don’t really feel like playing, my confidence just drops (Freddie).”

“If you’ve just had like a row with someone or your family you wouldn’t really want to be there because you know you’re not going to play as well if you’re, like, confident (Ashley).”

#### 3.2.5. Innate Factors

Innate factors mostly comprised of players having a good mentality for soccer, with a good mind-set giving them confidence to train and compete. Dan stated:

“You have to like start again because you might be a bit disappointed but if you stay disappointed you will just keep on losing, you gotta try and like boost so you can have confidence on the field.”

#### 3.2.6. Experience

Four players identified experience to be a source of self-confidence. This included playing experience and knowledge of teammates and the academy. It became apparent that becoming acquainted with aspects of the club was a determinant in confidence level for some performers, with Felix stating, “starting to know everyone and just starting playing up has just like increased my confidence a lot”.

#### 3.2.7. Athlete Specific Factors

These were factors that were unique to individuals and their confidence levels. This included role models performing well, seeing others fail, impression of opponents, crowd criticism, referee decisions, decision making in game, physical inferiority, situational favourableness, opposition comments and environment comfort.

## 4. Discussion

The present study represents the first attempt to examine the sources and types of confidence salient to academy football players, aged 11–12. We predicted that whilst Vealey and Chase’s [8] model and previous research [9,11] would provide a useful base from which to view our results, we would see differences in the types and sources of confidence deemed important to the performers due to maturational and environmental factors. Overall, this prediction was broadly supported. Our interpretations of the data do fit reasonably well into the dimensions generated in previous research but notable differences emerged in the lower- and higher order themes that contributed to these global dimensions.

In terms of the types of confidence, we found support for Vealey and Chase’s [8] contention of the multi-dimensionality of sport confidence and also found support for some of the dimensions seen in previous research. What is striking about our data is the relatively low quantity of raw data themes that emerged in the discussions with the participants. Whilst the need to be confident in their psychological factors (3 participants), superiority to the opposition (3 participants), tactical awareness (2 participants) and athlete specific factors (2 participants) was evident, the majority of the participants focused on either skill execution or achievement (confidence in their ability to gain selection/a continued contract). This is in contrast to the findings of Hays et al. [9] but is in line with the limited types of confidence identified by Thomas et al. [11]. In addition, a number of athletes found difficulty identifying their types of confidence. There are a number of reasons why this finding may have been seen. First, as Vealey et al. [7] noted, youth athletes are developing their own self-confidence and so the rather narrow types of confidence deemed important to the participants may reflect developing perception of competence and the determinants of success. Second, theory and research [12] within the achievement goal literature suggests that children may not develop a mature concept of ability and the ability to differentiate the determinants of success (e.g., the relative roles of luck and ability) until they are at least 11. Thus, the current findings may reflect a relative lack of knowledge of, or the ability to differentiate between, different determinants of performance.

In terms of confidence sources, we were able to categorise the responses of the participants into a number of the dimensions that have been identified in previous research. However, in contrast to the sources of confidence reported by elite, adult athletes [9], participants did not mention competitive advantage, trust, or self-awareness. Further, although our findings were in line with Thomas et al.’s [11] findings showing that the youth soccer players did seem to rely heavily on accomplishments, preparation and social support, the players in our research seemed to feel that the coach was a particularly powerful source of confidence. However, differences between the lower- and higher-order themes that emerged in our and previous research were more notable. First, whilst performance accomplishments were mentioned by all athletes in the Hays et al. paper, and all athletes in the current research, the range of responses in our study seemed to be less wide-ranging. From our analysis, participants relied on tangible indicators of accomplishment, such as goals scored/assisted and man of the match awards. This is consistent with the broader developmental literature which would suggest that the primary sources of competence information used by children in the childhood years (7–12 years old) are concrete in nature [4].

Such reliance on concrete sources of information is also consistent with the importance attached to social support and significant others. In our study, the information and feedback provided by coaches was identified by all participants as being influential in determining their confidence. Interestingly, our findings concerning the specific impact of coaching seems to be qualitatively different to the way in which coaches informed the confidence of participants in Hays et al. [9]. In Hays et al.’s research, lower order themes were more concerned with general support and belief in the coaches’ ability than with the provision of praise and criticism as seen in this study. From a development perspective, coach feedback represents a concrete source of information (Horn, 2004). Equally, the environment of the elite youth soccer academy is one in which selection is an ever-present stressor [21] and coaches are the people who make such decisions. As Will commented, “at the end of the season, they’re the ones who are going to decide whether I stay or go”.

A further difference from our results and both Hays et al.’s [9] and Thomas et al.’s [11] research is the role of holistic preparation and general mood as a determinant of confidence. In Hays et al.’s research, holistic preparation seems to refer predominantly to the perception that training had gone well whilst Thomas et al. focused on the practical impact of good preparation (e.g., tactical awareness, sound nutrition). Whilst we saw this within the current data, confidence was also influenced by general mood prior to competition or training. This can be seen through references to the moods and through references to the impact of external events such as events in the family or at school. Equally, the perception that family and team “atmospheres” could be interpreted as evidence of how an individual’s confidence may be influenced by their general feelings of contentment and happiness. The importance of mood resonates with Bandura’s [5] theorising concerning the role of emotional states as a source of efficacy beliefs. Specifically, Bandura argues that mood may influence perceptions of self-efficacy in a number of ways. First, negative life events and depressed mood may activate feelings of personal inadequacy and low self-worth, which may contribute to lower perceptions of one’s own competence. Second, negative moods activate memories of past failings, thus creating more negative self-efficacy, whilst a positive mood will activate memories of past successes, thus creating more positive self-efficacy. Finally, Bandura proposes that moods can influence the way in which events are evaluated so that mood may influence perceptions of the demands of the task at hand as well as perceptions of the resources available to oneself. Whilst our findings do not shed light on the ways in which mood may influence perceptions of confidence, they do suggest that the potential impact of mood on self-confidence should not be underestimated when working with children.

### 4.1. Applied Implications

There are two broad directions to take when considering the applied implications of this research. First, we can consider how best to enhance confidence by working through the sources of confidence known to be most salient for the client population [8]. In this instance, coaches and parents should be mindful of the role of concrete performance accomplishments, coaching feedback, peer and parental support and general mood/affect in determining confidence. That is, our findings first suggest that structuring practices and drills to provide the opportunity for concrete performance accomplishments and being mindful that feedback, particularly the beneficial effects of positive feedback, could be one way for coaches to enhance the confidence of the children they coach. A second suggestion that stems from our findings is to consider ways in which positive moods can be induced in youth performers before, during and after competition and training. This may be achieved through the actions of coaches and/or parents to provide activities that are enjoyed by the participants and are designed to lift moods or through the systematic use of positive psychology interventions such as the practice of grateful thinking or visualising best possible selves [22].

The second intervention option is to change the range of sources of confidence that are salient for the individual, so that (a) performers draw confidence from a range of sources and (b) performers become more reliant on controllable sources of confidence, such as mental preparation [8]. The results of this study suggest that young performers may be most reliant on the information provided by others and on the achievement of concrete performance accomplishments. Self-awareness and the ability to self-reflect and self-evaluate have been proposed [23,24] to be essential to the development of strong and stable perceptions of personal efficacy/confidence and have also been implicated in the development of elite level soccer players [25]. Thus, any strategy that develops self-reflection and self-evaluation, such as performance evaluation sheets [26], may be worthwhile.

### 4.2. Limitations

Even though this study provides an insight into the types and sources of confidence of elite level youth athletes, it does have limitations. First, the sample was, due to access issues, limited to one soccer academy. Given that Vealey and Chase [8] acknowledge that context and culture will influence sources of confidence then it is unclear the extent to which the current findings reflect the broader population of young soccer players or the context of this specific academy. Further research is needed to confirm these findings in a broader sample. Second, again mainly due to access and ethical reasons, we were not able to mitigate against some threats to validity as identified by critical realist researchers [18]. Specifically, although the lead researcher had completed a period of supervised experience within the academy, they had not prolonged engagement within the academy setting to collect triangulating data (e.g., observations, interviews with coaches/parents) or to build rapport and trust with the performers that may have led to richer data.

## 5. Conclusions

The sources of confidence identified by the athletes in this study broadly supported previous literature [8,9,11], but some notable differences were observed. Specifically, the young, male, elite-level, soccer performers interviewed provided evidence to suggest a relatively narrow range of types of confidence used (possibly indicative of a less mature understanding of the determinants of performance) and a reliance on more concrete sources of information such as tangible performance results (goals scored, goals saved, assists) and feedback from others (coaches predominantly but also family members and team-mates). We also saw the potential importance of general mood/affect in determining confidence levels. These results support the idea that sources of confidence are not universal and that they may be influenced by internal factors such as maturation levels and external factors such as coaching climate.

## Figures and Tables

**Table 1:** Numbers of participants referring to specific confidence types.

Type of Confidence	Number of Athletes Citing Type. *N* = 11	Total % of Athletes	Total % Citing in Hays et al. (2007) *N* = 14	Total % Citing in Thomas et al. (2021) *N* = 28
Skill Execution	9	82%	71%	100%
Achievement	6	64%	71%	--
Outcome	--	--	64%	--
Performance	--	--	29%	--
Physical Factors	--	--	64%	61%
Psychological Factors	3	27%	57%	46%
Superiority to Opposition	3	27%	50%	--
Tactical Awareness	2	18%	14%	--
Athlete Specific Factors	2	18%	50%	--

**Table 2:** Numbers of participants referring to specific confidence sources.

Source of Confidence	Number of Athletes Citing Source (*N* = 11)	Total % of Athletes	Total % Citing in Hays et al. (2007)	Total % Citing in Thomas et al. (2021)
**Preparation**	9	82%	100%	82%
Physical preparation	7	64%	100%	75%
Mental preparation	1	9%	76%	50%
Holistic preparation	7	64%	57%	14%
**Performance Accomplishments**	11	100%	100%	100%
Competition accomplishments	11	100%	100%	96%
Training accomplishments	9	82%	50%	96%
Other accomplishments	4	36%	--	64%
**Coaching**	11	100%	93%	--
Individual interactions with coach	11	100%	--	--
Interactions with coach in group	3	27%	--	--
**Social Support**	10	91%	57%	100%
Family	7	64%	--	--
Team-mates	9	82%	--	--
School/Friends	3	27%	--	--
Other	2	18%	--	--
**Innate Factors**	4	36%	50%	29%
**Experience**	4	36%	43%	--
**Athlete Specific Factors**	4	36%	50%	--
**Competitive Advantage**	--	--	36%	--
**Trust**	--	--	14%	--
**Self-Awareness**	--	--	14%	--

## Data Availability

Data not available due to ethical restrictions—Due to the nature of this research, participants of this study did not agree for their data to be shared publicly, so supporting data is not available.

## Supplementary Materials

The following are available online at https://www.mdpi.com/article/10.3390/sports9110146/s1, Table S1: Global dimensions and themes for types of confidence, Table S2: Themes and categories for sources of confidence identified by the academy youth athletes.
