# Supplementary material for: Elite Youth Soccer Players’ Sources and Types of Soccer Confidence
Source: Sports (Basel). 2021 Oct 22;9(11):146. doi: 10.3390/sports9110146 (PMC8617682; doi:10.3390/sports9110146)
Supplement: Supplementary file 1 [file sports-09-00146-s001.zip › sports-1368768-supplementary.pdf]

sports MDPI

Table S1. Global dimensions and themes for types of confidence (numbers in parentheses illustrate the number of participants citing the source when >1).

Raw Data Themes.	Higher Order Themes	Global Dimension
Winning	Outcome	ACHIEVEMENT
Scoring goals (3)<br>Confidence to play well	Performance	
Passing (4)<br>Passing in the final third (1)	Passing	SKILL EXECUTION
Shooting (2)<br>Tackling (3)<br>Keeping possession<br>Using both feet<br>Body movement<br>Receiving the ball<br>Dribbling	General Skills	
Coming back from mistakes<br>Attitude<br>Handling pressure<br>Mentality		PSYCHOLOGICAL FACTORS
Beating other players<br>Playing against weaker teams<br>Knowing opposition's play		SUPERIORITY TO OPPOSITION
Good judgement<br>Decision making		TACTICAL AWARENESS
Communication		ATHLETE SPECIFIC FACTORS

Table S2. Themes and categories for sources of confidence identified by the academy youth athletes (numbers in parentheses illustrate the number of participants citing the source when >1).

Raw Data Themes	Sub-Themes	Lower Order Themes	Higher Order Themes	Global Dimensions
Good condition<br>Injury status (2)<br>Muscles feeling good (2)<br>Warming up properly (2)<br>Being rested (2)		Physical State	Physical Preparation	PREPARATION
Having your 5 a day<br>Eating breakfast<br>Eating well		Diet		
Looking good		Self-presentation		

- Mental Preparation
  - Imagery
- Holistic Preparation
  - Being in a good mood (3)
  - School experiences before a match (4)
  - Being late (2)

- PERFORMANCE ACCOMPLISHMENTS
  - Training Accomplishments
    - Skill Development
      - Mastering/not mastering a skill (6)
      - Knowing a skill works
      - Working on different skills
    - General Training Performance
      - Good training sessions (6)
      - Executing skill in training
      - Correcting mistakes in training
      - Working hard
    - Outperforming Team-mates
      - Playing well against superior/older opposition (3)
      - Being superior/inferior to teammates (3)
      - Tackling someone bigger
  - Competition Accomplishments
    - Demonstration of Ability
      - Win/Loss
        - Beating superior opposition (3)
        - Losing (2)
        - Losing against a side ranked below you
        - Winning consecutive games
      - Selection/Awards
        - Winning a trophy
        - Winning man of the match
    - Performance
      - Scoring (8)
      - Playing well/poorly (6)
      - Making good passes (4)
      - Keeping/not keeping the ball (3)
      - Ball placement
      - Creating goals
      - Losing tackles
      - Making mistakes
      - Bad first quarter
      - Saving a goal
  - Other Accomplishments
    - Beating school classmates (3)
    - Doing well in cricket
    - Doing well at school

Raw Data Themes	Sub-Themes	Lower Order Themes	Higher Order Themes	Global Dimensions

- Recognition
  - Being selected (2)
  - Being asked to train/play with higher age groups (3)
  - Signing a contract (2)
  - Selected to play preferred position
  - Praise from another coach
  - Selected to be captain
  - Award for training
  - Receiving praise (11)

Not receiving praise (2)<br>Criticism<br>Coach moaning (4)<br>Others receiving praise from coach		Praise	Individual interactions with coach	COACHING
General feedback on progress (3)<br>Coach encouragement and reassurance (5)<br>Encouragement to try new skills<br>Positive criticism<br>Finding time for players		Attention and Encouragement		
Treating all players equally<br>Favoritism<br>Interactions with support staff			Interactions with coach in group	
Arguments<br>Getting along with family<br>Family outings<br>Family illness		Atmosphere	Family Support	SOCIAL SUPPORT
Negative feedback (2)<br>Positive feedback (3)<br>Honest opinion<br>Encouragement after poor games		Feedback		
Family attending matches<br>Family giving advice<br>Encouragement before a game<br>Family treats<br>Traveling with family<br>Encouragement during a game<br>Asking about training		Support		
Being left out of activities by friends<br>Fun with friends<br>Playing football with friends<br>Teacher encouragement			Friends/School Support	
Intimidating crowd<br>Spectator praise			Other Support	
**Raw Data Themes**	**Sub-Themes**	**Lower Order Themes**	**Higher Order Themes**	**Global Dimensions**
Positive banter<br>Negative banter<br>Team atmosphere<br>Team-mate pranks<br>Being mean after the game<br>Team getting annoyed<br>Unenthusiastic team-mates		Team atmosphere	Team-mate Support	SOCIAL SUPPORT (Cont'd)
Positive feedback (2)<br>Praise (5)	Positive feedback	Feedback		
Team-mates moan at me (5)<br>Team questioning my decisions	Negative feedback			

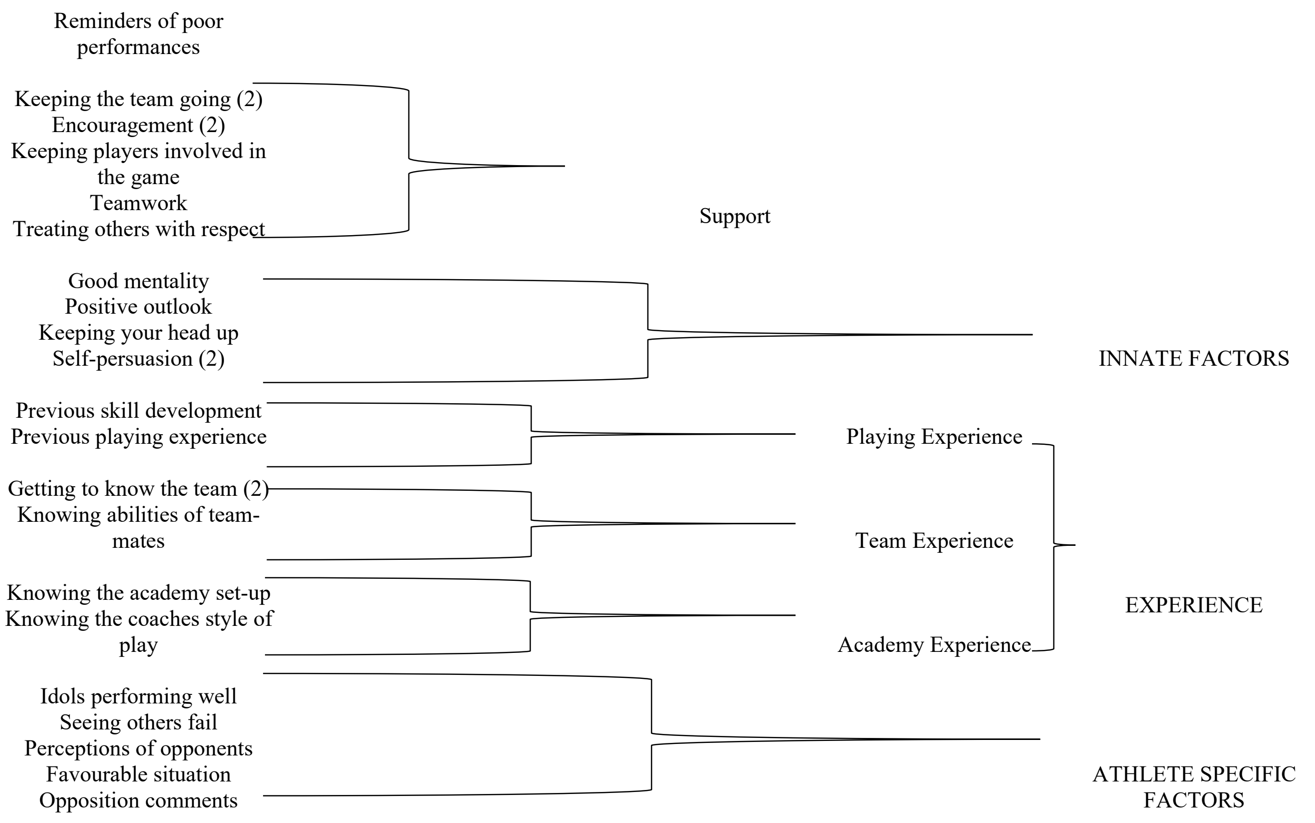
Reminders of poor performances
Keeping the team going (2)
Encouragement (2)
Keeping players involved in the game
Teamwork
Treating others with respect
Support
Good mentality
Positive outlook
Keeping your head up
Self-persuasion (2)
INNATE FACTORS
Previous skill development
Previous playing experience
Playing Experience
Getting to know the team (2)
Knowing abilities of team-mates
Team Experience
Knowing the academy set-up
Knowing the coaches style of play
Academy Experience
EXPERIENCE
Idols performing well
Seeing others fail
Perceptions of opponents
Favourable situation
Opposition comments
ATHLETE SPECIFIC FACTORS
